# History of academic family medicine in South Africa – When did it start?

**DOI:** 10.4102/safp.v62i1.5031

**Published:** 2020-03-25

**Authors:** Bernhard Gaede

**Affiliations:** 1 Department of Family Medicine, School of Nursing and Public Health, University of KwaZulu-Natal, Durban, South Africa

**Keywords:** family medicine, history, COPC, academic medicine, SAAFP

## Abstract

The literature on the history of family medicine as an academic discipline locates its beginning with the establishment of two faculties linked to the Royal College of General Practice in 1958. However, the history of Community Oriented Primary Care documents, how the Kark’s moved from Pholela in KwaZulu-Natal, were involved with the establishment of the Natal Medical School in Durban. As part of this a Department of Social, Preventative and Family Medicine was established in 1956 with Dr Sidney Kark as its first Head of Department. The South African Academy of Family Practice and Primary Care established in 1980 explicitly orientated itself in relation to public healthcare (PHC). We need to re-claim the history of Community Oriented Primary Care as part of the history of family medicine and proudly trace our current ethos and values to the seminal work of the Kark’s.

In South Africa, general practitioners have been the backbone for providing generalist care to the majority of the population. Historically, the medical officers in clinics and hospitals, the private practitioners in general practice all over the country as well as the mission doctors essentially providing the core of health care services in many rural areas predated generalist care as a distinct academic discipline.^1^

The literature on the development of family medicine as an academic discipline in South Africa mostly refers to an address by Dr Basil Jaffe,^2^ the first president of the South African Academy of Family Practice and Primary Care (SAAFP) (see, for instance, Hugo and Allan,^1^ Hellenberg et al.^3^ and Williams and Reid^4^). Key high-level milestones in Dr Jaffe’s account locate the beginning of academic family medicine in 1958 when the Cape of Good Hope and Witwatersrand regions formally established faculties linked to the Royal College of General Practitioners in the United Kingdom, the first appointment of a professor of family medicine in 1967 at the University of Pretoria and the establishment of the SAAFP in 1980. I mention these three milestones specifically in relation to a different history related to family medicine.

The body of literature around community-oriented primary care (COPC) documents how Sidney and Emily Kark together with Edward Jali (a health educational practitioner) and others are credited for their pioneering work at Pholela in KwaZulu-Natal in the 1940s and how this contributed to the establishment of primary health care (PHC) (see, for instance, Williams and Reid,^4^ Longlett et al.,^5^ and Marcus^6^). What is less well-known is how the COPC initiatives were explicitly linked with the establishment of academic family medicine in South Africa. In her book on the history of the medical school in Durban, Vanessa Noble^7^ narrates how in the early 1950s, as part of the establishment of the Natal Medical School, the then Dean George Gale and Sidney Kark convinced the faculty board to establish Family Practice and Community Medicine as a main clinical division alongside Medicine Surgery and Obstetrics and Gynaecology (p. 76). When the Natal Medical School opened in 1956, the name was changed to the ‘Department of Social, Preventative and Family Medicine’ (p. 77), and Sidney Kark was appointed as the first professor and head of department. The clinical training of medical students in family medicine took place at the Institute of Family and Community Health (IFCH) – a network of community health centres throughout Durban which were based on the COPC model pioneered in Pholela.^6^ This was akin to what is currently referred to as a ‘decentralised training platform’. The community health centres were linked with the communities through community-level health promoters as part of the COPC model, which resonates with current initiatives of re-engineering PHC in South Africa.^4^ Sadly, both the Department of Social, Preventative and Family Medicine at the University and the IFCH were dissolved owing to complex national and professional politics prevalent in the 1950s and early 1960s, which were profoundly antagonistic to such innovative approaches at that time.^6,7^

When the SAAFP was formed in 1980, it included PHC as a core aspect of family medicine,^2^ which has subsequently become the central focus in the training of family physicians.^3^ The founding documents quoted the Alma Ata Declaration as the SAAFP’s orientation and thereby essentially returning to the path that the Karks and their colleagues had shown in Pholela and Durban. We can but marvel how visionary the Karks were – not only in terms of development of COPC but also with regard to establishing academic family medicine and including it in undergraduate medical education.

We need to reclaim our history, not only to acknowledge the work of those before us but also to proudly trace our current ethos and values to their roots. It links our current efforts to the seminal work conducted at the Pholela Health Centre, the birthplace of COPC.
